# Cancer burden attributable to human papillomavirus infection by sex, cancer site, age, and geographical area in China

**DOI:** 10.1002/cam4.2697

**Published:** 2019-11-12

**Authors:** Rufei Duan, Youlin Qiao, Gary Clifford, Fanghui Zhao

**Affiliations:** ^1^ National Cancer Center National Clinical Research Center for Cancer Cancer Hospital Chinese Academy of Medical Sciences and Peking Union Medical College Beijing China; ^2^ International Agency for Research on Cancer Lyon France

**Keywords:** attributable fraction, burden, cancer, China, human papillomavirus

## Abstract

**Background:**

Human papillomavirus (HPV) attributable cancer burden is currently unknown in China, which is essential to evaluate the potential benefit of existing HPV https://www.sciencedirect.com/topics/medicine-and-dentistry/vaccine and to inform cancer control policy.

**Methods:**

We extracted data of cancer incidence and mortality in 2014 from Chinese Cancer Registry Annual Report, and data of national population from National Bureau of Statistics. HPV‐attributable cancer burden was estimated by incorporating cancer rates and population forecasts by sex, cancer site, age and geographical area, and then combined to corresponding population attributable fractions.

**Results:**

We estimated that there were 110 894 HPV‐attributable new cancer cases in China in 2014, including 99 253 cervical cancers, 4449 noncervical cancers in females and 7192 cancers in males. The age‐standardized incidence of HPV‐attributable cancers in China was 5.69 per 100 000 persons, being slightly higher in rural than urban areas. Specifically, 51.1% of HPV‐attributable cervical cancers were diagnosed in women aged 40‐54 years, while 75.8% of noncervical cancers were diagnosed at 45‐79 years of age. Among males, 53.4% of cancers were diagnosed at 55‐74 years of age. Thirty five thousand six hundred and eighty three HPV‐attributable cancer deaths were estimated, including 29 683 due to cervical cancer, and 2307 and 3693 due to noncervical cancer in females and males, respectively.

**Conclusions:**

The cancer burden attributable to HPV in China is substantial. HPV vaccination and cervical screening should be prioritized.

## Graphical abstract

Human papillomavirus (HPV) attributable cancer burden is currently unknown in China; we estimated HPV‐attributable cancer cases and deaths by sex, cancer site, age and geographical area. There is a pressing need for these data to inform evidence‐based policy with regard to HPV vaccination and screening programs.

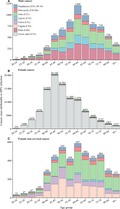

## 1 INTRODUCTION

Oncogenic human papillomavirus (HPV) infection, especially HPV 16/18, is causally related to not only cervical cancer, but also other anogenital cancers of the vulva, vagina, penis, anus, as well as head and neck cancers.^1^ It was estimated that 630 000 (4.5%) cancer cases worldwide in 2012 were attributable to HPV infection with 570 000 among females and 60 000 among males.^2^ Globally, cervical cancer contributed 530 000 new cases per year, accounting for 84% of all HPV‐attributable cancers, while the other anogenital cancers, head and neck cancers combined contributed around 100 000 cancer cases (16%).^2^

Cervical cancer has been successfully prevented by screening in some developed countries,^3^, ^4^ and will be further impacted by HPV vaccination. Meanwhile, HPV‐attributable noncervical cancers have attracted more and more attention. Increasing trends of incidence of cancers of anus, oropharynx, penis, vulva and vagina have been reported in several high income countries, such as UK, USA, Italy, Norway and Canada.^5^, ^6^, ^7^, ^8^, ^9^, ^10^ With respect to prevention at these noncervical sites, for which there were no effective screening programs, the prophylactic HPV vaccine has been demonstrated to have an excellent protective effect against HPV infection and/or lesions.^11^, ^12^, ^13^ Furthermore, compared to cervical cancer, HPV16 had a greater predominance in anal cancer as well as head and neck cancer.^2^ HPV 16 and 18 together are responsible for 87% and 85% of anal cancer and head and neck cancer, respectively.^2^ This indicates the potential benefit of the three existing prophylactic https://www.sciencedirect.com/topics/medicine-and-dentistry/vaccine against HPV types 16 and 18. During the last decades, over 80 countries (the majority being high or upper‐middle income) have introduced HPV vaccination into the national immunization programs,^14^ and are expected to benefit greatly.

Appropriate HPV‐attributable cancer control strategies regarding primary and secondary prevention are based on comprehensive understanding of the disease burden. However, burden of HPV‐attributable cancers in China is not well‐characterized. The Chinese government is now prioritizing population health, and has made several considerable health investments and promotion actions, such as conditional approval of the nonavalent HPV vaccine following introduction of bivalent vaccine and quadrivalent vaccine in mainland China.^15^ Understanding the burden of HPV‐attributable cancer can boost not only programs of HPV vaccination but also HPV‐based cancer screening in China, and can provide basic data for cost‐effective evaluation of these intervention programs. Moreover, it is essential to monitor trends in HPV‐attributable cancer incidence that might result from future increasing use of HPV vaccines and changes in cervical cancer screening practices. In this work, we calculated the cancer burden attributable to HPV infection for the year 2014 by cancer site, sex, geographical area and age.

## 2 MATERIALS AND METHODS

HPV‐attributable cancers are defined as cancers of cervix uteri (C53), anus (C21), vulva (C51), vagina (C52), penis (C60), oropharynx (C01, 09‐10), oral cavity (C02‐06), larynx (C32), and other pharynx (C12‐C14), according to the findings from International Agency for Research on Cancer (IARC) Monographs.^1^

### 2.1 Data sources

Age‐specific rate of cancer cases and deaths was obtained from the annual report of Chinese cancer registry published in 2018.^16^, ^17^ Since the typical population‐based cancer registry has three years of time lags from cancer incident to data collection, the data we extracted was actually the cancer cases or deaths occurred in 2014.^16^, ^17^ In 2014, 449 cancer registries (160 in urban areas and 289 in rural areas) from all 31 provinces in mainland China submitted cancer registry data to the National Central Cancer Registry.^16^, ^17^ These cancer registries covered 346 million persons (176 males and 170 females), accounting for about 25.27% of the national population.^16^, ^17^ All submitted cancer registry data underwent quality control based on the criteria of “Guideline for Chinese Cancer Registration” and “Cancer Incidence in Five Continents Volume IX”.^16^, ^17^ After data quality control, data from 339 cancer registries met the criteria and were included in this study, of which 129 located at urban areas and 210 at rural areas.^16^, ^17^ These qualified cancer registries covered 288 million persons (146 males and 142 females), accounting for about 21.07% of the total national population.^16^, ^17^

Age‐specific population size was estimated using the data released by the National Bureau of Statistics of China.^18^ First, the changes in age‐specific death probabilities between 2000 and 2010 were calculated by referring the data from the fifth and sixth National Population Census.^19^ Second, we estimated age‐specific death probabilities in 2011‐2014 with the assumption of a linear trend from 2000 to 2014.^20^ Third, the estimated death probabilities from 2011 to 2014 were used to calculated the age structure of the population in 2014.^20^ Finally, the age‐specific population size was calculated using the estimated age structure in 2014 and the national total population of 2014 released by the National Bureau of Statistics.^20^

### 2.2 Statistical analyses

The national estimates of HPV‐associated cancer cases and deaths were calculated by multiplying age‐specific rates with the estimated population size. All the estimates were stratified by sex, cancer site, age, and geographical area for cancer cases and deaths separately.^16^ Cancers attributable to HPV were calculated by multiplying site‐, sex‐, area‐, and age‐specific HPV‐associated cancer burden with corresponding population attributable fractions (PAFs).^21^, ^22^ Segi's population was used to calculate age‐standardized rate. The corresponding estimates for 95% confidence interval (CI) of the HPV‐attributable cancers were calculated from the lower and upper bounds of age‐specific PAFs, which were estimated using a bootstrap simulation method with 5000 simulations.^21^ The total number and 95% CI of attributable cancers in males and females for each cancer site were then calculated by summing across all age groups, respectively.

## 3 RESULTS

### 3.1 HPV‐attributable cancer cases and incidence

We estimated 165 914 cancer cases at anatomical sites linked with HPV infection per year in China. Among these, 110 894 were causally attributable to HPV infection, including 7192 (6.5%) male cancers, 99 253 (89.5%) cervical cancers, 4449 (4.0%) female non‐cervical cancers (Table 1). The noncervical cancers in descending order among females were anus (1678), vagina (1237), vulva (736), oropharynx (342), oral cavity (336), larynx (120), and among males were cancers of the penis (2392), anus (2258), oropharynx (1032), larynx (955), and oral cavity (555) (Table 1). Urban and rural areas contributed 61 463 and 49 431 total cancer cases respectively, but rural areas had slightly higher age‐standardized incidence rate (ASIR) of total HPV‐attributable cancers (5.94 vs 5.52 per 100 000 persons) and cervical cancer (10.99 vs 10.01) than urban areas (Table 1). ASIR of noncervical cancers was similar between rural and urban areas (0.57 vs 0.53), and there was no obvious disparity of ASIR for individual noncervical cancer sites (Appendix S1).

**Table 1:** Cancer cases and incidence (per 100 000 persons) attributable to HPV infection in China, 2014

Cancer site (ICD‐10)	Total				Male			Female		
	Cases	PAF (%)^21^, ^22^	Cases attributable to HPV	ASIR	Cases	Cases attributable to HPV	ASIR	Cases	Cases attributable to HPV	ASIR
All areas										
Cervix uteri (C53)	102 074	97.4	99 253	5.15	—	—	—	102 074	99 253	10.42
Anus (C21)	4493	88.0	3936	0.17	2575	2258	0.20	1918	1678	0.15
Vulva (C51)	2789	24.1	736	0.04	—	—	—	2789	736	0.07
Vagina (C52)	1601	78.0	1237	0.06	—	—	—	1601	1237	0.12
Penis (C60)	4700	51.0	2392	0.11	4700	2392	0.22	—	—	—
Oropharynx (C01, 09‐10)	6018	23.0	1374	0.07	4516	1032	0.10	1503	342	0.03
Oral cavity (C02‐06)	20 831	4.3	891	0.04	12 954	555	0.05	7876	336	0.03
Larynx (C32)	23 408	4.6	1075	0.05	20 786	955	0.09	2622	120	0.01
Total	165 914	—	110 894	5.69	45 531	7192	0.66	120 383	103 702	10.83
Urban areas										
Cervix uteri (C53)	56 446	97.4	54 875	4.99	—	—	—	56 446	54 875	10.01
Other HPV‐attributable cancers	39 285	—	6588	0.53	28 138	4023	0.64	11 147	2564	0.40
Total	95 731	—	61 463	5.52	28 138	4023	0.64	67 593	57 439	10.41
Rural areas										
Cervix uteri (C53)	45 628	97.4	44 377	5.37	—	—	—	45 628	44 377	10.99
Other HPV‐attributable cancers	24 555	—	5054	0.57	17 393	3170	0.71	7162	1885	0.43
Total	70 183	—	49 431	5.94	17 393	3170	0.71	52 790	46 262	11.42

Abbreviations: ASIR, age‐standardized incidence rate; HPV, human papillomavirus; ICD‐10, International Classification of Diseases 10th revision; PAF, population attributable fraction.

**Figure 1:**
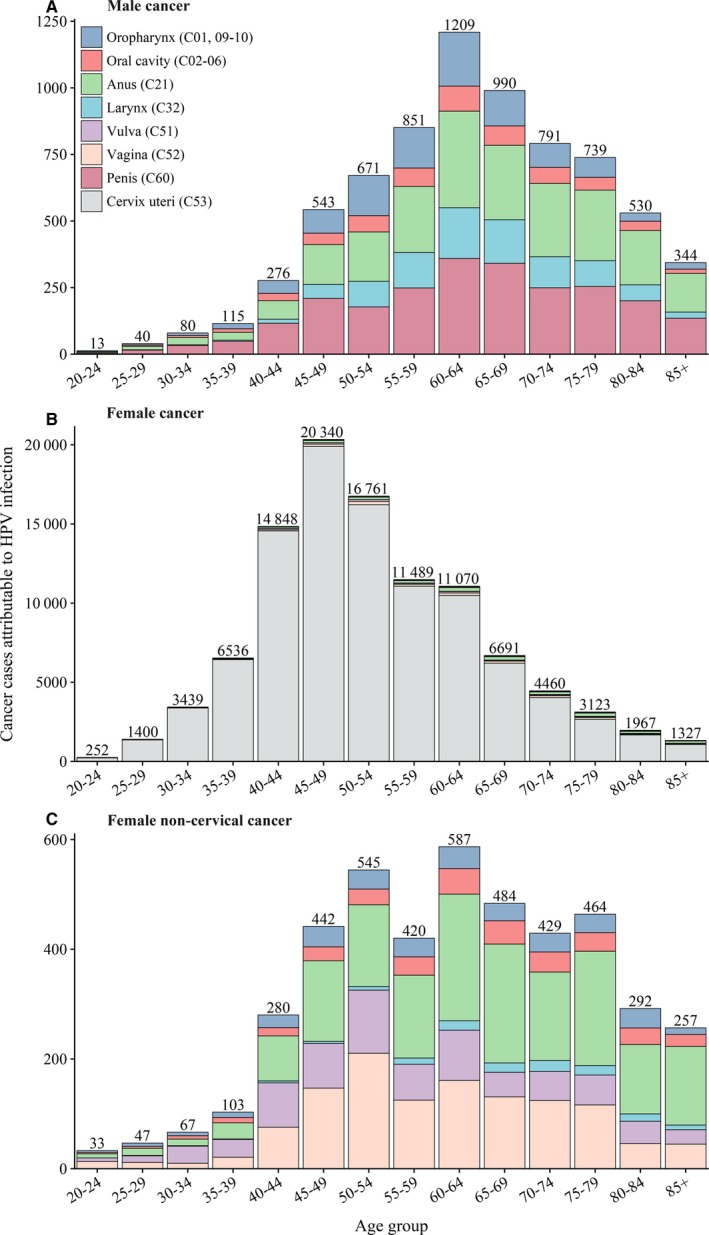
Age‐specific cancer cases attributable to HPV infection in China, 2014. HPV, human papillomavirus

**Figure 2:**
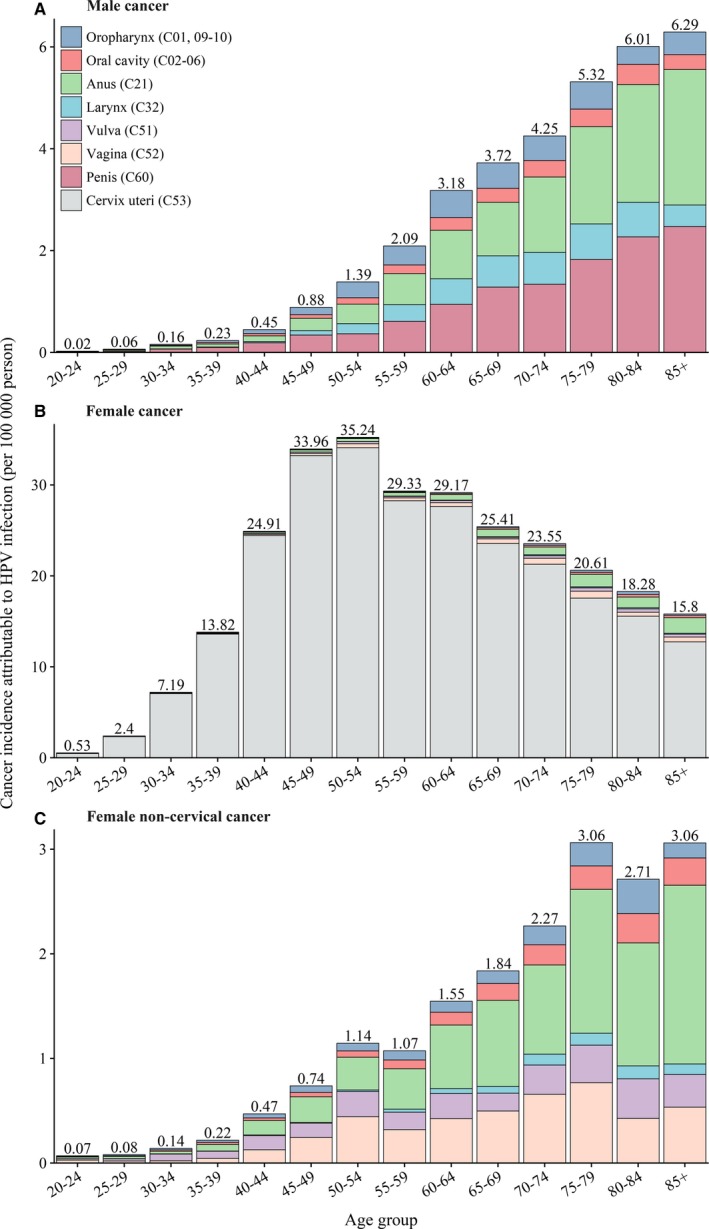
Age‐specific cancer incidence attributable to HPV infection in China, 2014. HPV, human papillomavirus

Among females, 51.1% of HPV‐attributable cervical cancers were diagnosed in women aged 40‐54 years with the peak among those aged 45‐50 years, while 75.8% of noncervical cancers were diagnosed in women within the plateau age intervals of 45‐79 years (Figure 1). Among males, 53.4% of HPV‐attributable cancers were diagnosed in men aged 55‐74 years with the peak among those aged 60‐64 years (Figure 1). The ASIR of all cancers combined was 5.69 per 100 000 persons in the total population, with 10.83 and 0.66 in females and males, respectively (Table 1). Among females, a rapid rising trend of the ASIR for cervical cancer was observed up to a peaking at 45‐54 years old, with a slight decreasing trend hereafter (Figure 2). Whereas the ASIR for noncervical cancers among females and cancers among males increased continually with advancing age (Figure 2).

### 3.2 HPV‐attributable cancer deaths and mortality

In China, 63 183 cancer deaths were estimated related to HPV infection per year. A total of 35 683 cancer deaths were attributable to HPV infection, including 29 683 due to cervical cancer (83.2%), 2307 (6.5%) and 3693 (10.3%) due to noncervical cancer in females and males, respectively (Table 2). Among females, most common noncervical cancer deaths were, in descending order, anus (1150), vagina (533), vulva (248), oral cavity (159), oropharynx (139), and larynx (78) (Table 2). In males, anal cancer contributed the most cancer deaths (1623), followed by cancers of penis (745), larynx (529), oropharynx (511), oral cavity (285) (Table 2). Urban and rural areas contributed 19 492 and 16 192 HPV‐attributable cancer deaths, respectively, with the age‐standardized mortality rate (ASMR) being slightly higher in rural than in urban areas (1.79 vs 1.54 per 100 000 persons). Rural areas showed slightly higher ASMR of cervical cancer (3.14 vs 2.63) than urban areas while ASMR of noncervical cancers was similar (0.25 vs 0.23) (Table 2). There was no obvious disparity of ASMR for individual noncervical cancer sites (Appendix S1).

**Table 2:** Cancer deaths and mortality (per 100 000 persons) attributable to HPV infection in China, 2014

Cancer site (ICD‐10)	Total				Male			Female		
	Deaths	PAF (%)^21^, ^22^	Deaths attributable to HPV	ASMR	Deaths	Deaths attributable to HPV	ASMR	Deaths	Deaths attributable to HPV	ASMR
All areas										
Cervix uteri (C53)	30 464	97.4	29 683	1.41	—	—	—	30 464	29 683	2.84
Anus (C21)	3157	88.0	2773	0.11	1849	1623	0.13	1308	1150	0.08
Vulva (C51)	1043	24.1	248	0.01	—	—	—	1043	248	0.02
Vagina (C52)	693	78.0	533	0.02	—	—	—	693	533	0.04
Penis (C60)	1460	51.0	745	0.03	1460	745	0.06	—	—	—
Oropharynx (C01, 09‐10)	2825	23.0	650	0.03	2222	511	0.05	603	139	0.01
Oral cavity (C02‐06)	10 340	4.3	444	0.02	6627	285	0.03	3713	159	0.01
Larynx (C32)	13 201	4.6	607	0.03	11 509	529	0.05	1692	78	0.01
Total	63 183	—	35 683	1.66	23 667	3693	0.32	39 516	31 990	3.01
Urban areas										
Cervix uteri (C53)	16 429	97.4	16 003	1.31	—	—	—	16 429	16 003	2.63
Other HPV‐attributable cancers	19 894	—	3489	0.23	14 534	2138	0.30	5360	1350	0.16
Total	36 323	—	19 492	1.54	14 534	2138	0.30	21 789	17 353	2.79
Rural areas										
Cervix uteri (C53)	14 035	97.4	13 680	1.54	—	—	—	14 035	13 680	3.14
Other HPV‐attributable cancers	12 825	—	2512	0.25	9133	1555	0.30	3692	957	0.18
Total	26 860	—	16 192	1.79	9133	1555	0.30	17 727	14 637	3.32

Abbreviations: ASMR, age‐standardized mortality rate; HPV, human papillomavirus; ICD‐10, International Classification of Diseases 10th revision; PAF, population attributable fraction.

**Figure 3:**
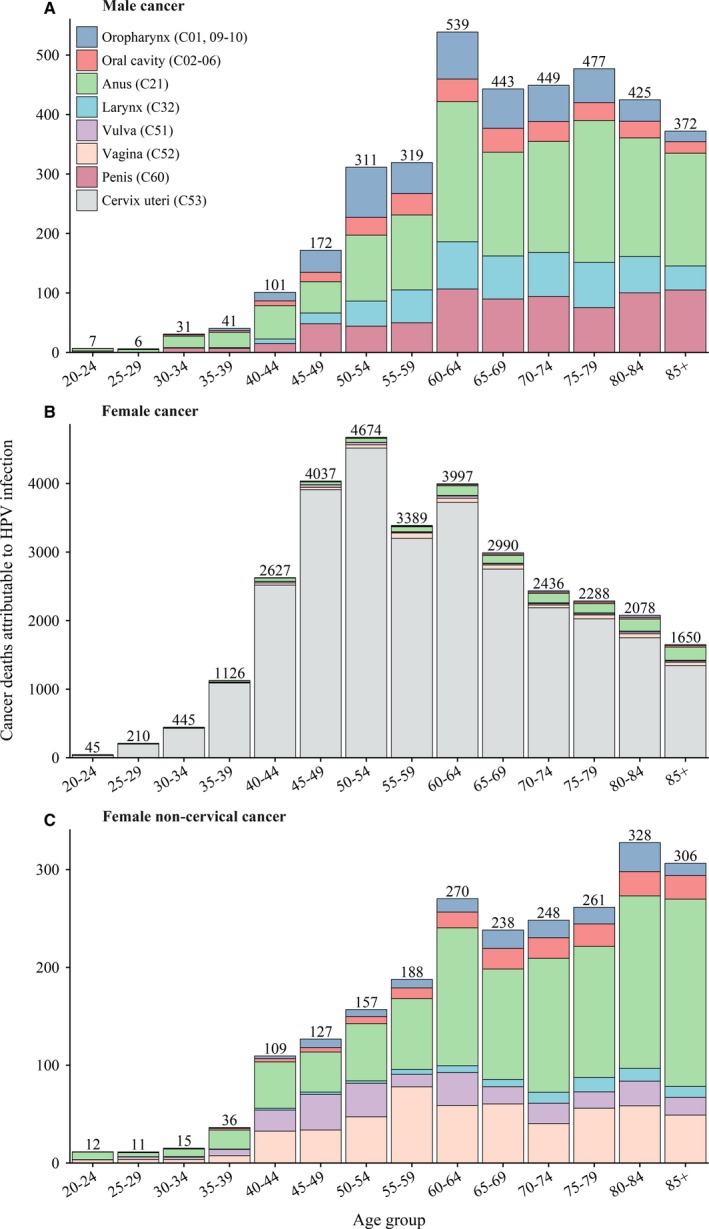
Age‐specific cancer deaths attributable to HPV infection in China, 2014. HPV, human papillomavirus

**Figure 4:**
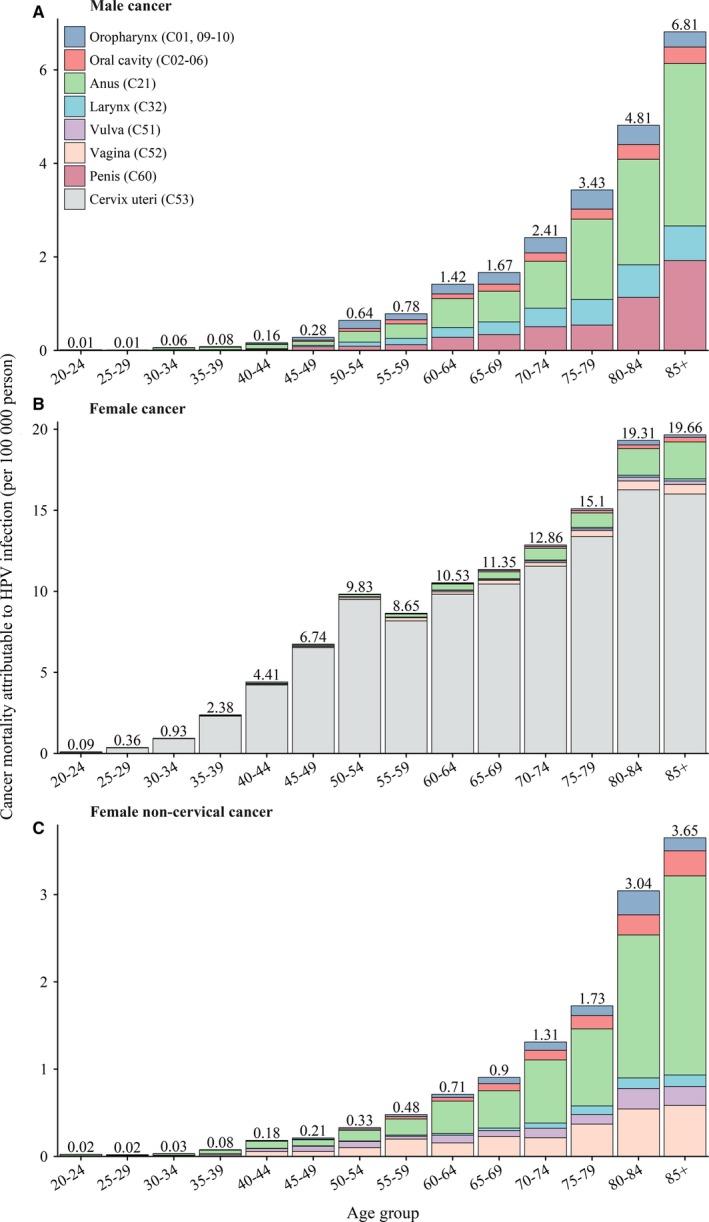
Age‐specific cancer mortality attributable to HPV infection in China, 2014. HPV, human papillomavirus

In females, 51.7% of cervical cancer deaths occurred among women within the age group 45‐64 years with the peak among those aged 50‐54 years (Figure 3). For noncervical cancer, 71.6% and 73.0% of deaths occurred after 60 years of age in females and males, with the peak among those aged 80‐84 years and 60‐64 years, respectively (Figure 3). The ASMR for all HPV‐attributable cancers was 1.66 per 100 000 persons, being 3.01 and 0.32 in females and males respectively (Table 2). The ASMRs for cervical cancer, female noncervical cancer and male cancers all showed a continuous increasing trend by age (Figure 4).

## 4 DISCUSSION

HPV‐attributable cancers were responsible for 110 894 cancer cases and 35 683 cancer deaths per year in the total population in China, accounting for 2.9% and 1.6% of the total nationwide burden of new cancer cases (3 804 000) and cancer deaths (2 296 000), respectively.^17^ Cervical cancer dominated HPV‐attributable cancer burden (89.5% of new cancers), while female noncervical cancers (4.0%) and male cancers (6.5%) constituted the remaining 10.5%.

In China, the top three cancer types attributable to HPV infection in total population were cancers of the cervix (99 253, 90%), anus (3936, 4%), and head and neck (3340, 3%). This compares to cervix (530 000, 84%), head and neck (37 200, 6%), anus (35 000, 6%) in global estimates.^2^ However, the HPV‐attributable cancer profile in China is markedly different from those of developed countries. During 2012‐2016 in the US, oropharyngeal cancer (12 600, 39%), cervical cancer (9700, 30%) and anal cancer (6000, 19%) were the three most common cancers.^23^ In China, females dominated HPV attributable cancers (93.5% in females vs 6.5% in males), because about 90% of HPV attributable cancers are cervical cancer. It was dramatically different from that in the US ( 59.5% in females vs 40.5% in males^23^), where cancer cases were driven by male oropharyngeal cancer. In some developed countries like the US, cervical cancer has been well‐controlled due to successful population‐based screening and vaccination programs. Along with the increasing trend of head and neck cancer and other anogenital cancers, the proportion of noncervical cancers is relatively large. Due to the lack of effective early screening and HPV vaccination coverage, other anogenital cancers and head and neck cancers may increase in the future in China. This is a potential key target for HPV‐attributable cancer control.

Males contributed 3.2 times head and neck cancers than that of females (2542 vs 798), and the number of cancer cases were much higher than that of previous assessment (950 vs 270).^2^ Similarly, a global study reported more HPV attributable head and neck cancers in males than that in females worldwide.^2^ We estimated more anal cancers in males than females (2258 vs 1678) in China, according to previous reported data (5900 vs 3600)^2^ derived from a limited number of registry sites from China.^24^ The male‐to‐female ratio was opposite to that seen globally^2^ and in specific reports from settings such as the US,^23^ Europe^25^ and Germany,^26^ showing that anal cancer was more common in females. However, in our study, anal and mis‐classified rectal adenocarcinomas that not linked to HPV infection were included because the pathological type is not reported in the Chinese cancer registry. This may lead to an overestimation of the total anal cancer cases attributable to HPV, particularly among males (in whom rectal cancer is more common). Indeed, a study in Liuzhou, China reported that Chinese females had higher incidence of anal HR‐HPV infection than males,^27^ indicating a probably higher anal cancer burden in females than males, even though the sex difference in the incidence of anal cancer in Liuzhou could not absolutely represent the overall sex trend of China. Precise estimation of HPV‐attributable anal cancer burden needs further histological classification.

Urban and rural areas represented about 55% and 45% total population in China, respectively,^20^ they correspondingly contributed 55.4% and 44.6% cancer cases and deaths attributable to HPV. However, rural areas showed a slightly higher ASIR and ASMR of cervical cancer than urban areas, in spite of the similar HR‐HPV prevalence in rural and urban areas,^28^ which may indicate a poorer screening access and lower screening coverage in rural areas. A study reported that lots of cases need further diagnosis and pre‐cancer treatment were failed to follow‐up in rural China.^29^ Furthermore, rural women were consistently less likely than women in urban areas to report having had a cervical cancer screening in China.^30^ It probably highlights the fact that rural areas need more government‐funded health resources and services to alleviate the conflict between relatively higher cervical cancer burden and lower accessible health care. There was no obvious discrepancy in ASIR and ASMR of noncervical cancers between rural and urban areas.

Cervical cancer carries the highest burden attributable to HPV at present in China, and more than half of new cervical cancer cases occur within the age group 40‐54. In order to reduce the incidence of cervical cancers without delay, population‐based vaccination and screen‐and‐treat programs should be accessible and available to the public. To be specific, HPV vaccination should be prioritized for Chinese adolescent girls. The screening technologies still need substantial improvements even though China has obtained certain achievements in fighting against cervical cancer by adopting several screening strategies.^31^ The Chinese government has made enormous investment on cervical cancer screening since 2009,^15^ but the national screening system still needs substantial improvement. Bivalent vaccine, quadrivalent vaccine and nonavalent HPV vaccines have been approved in 2016, 2017 and 2018, respectively in mainland China. However, there are still substantial obstacles for HPV vaccines being rolled out in China, and being included into the National Immunization Program^15^ to limit HPV‐attributable cancer control. More efforts still need to be taken to respond to WHO action call for eliminating cervical cancer.

Unlike cervical cancer, there are currently no standardized practices for routine screening for other HPV‐attributable noncervical cancers, but they would be well prevented by HPV vaccination.^32^, ^33^ A study illustrated that the vaccine efficacy against anal HPV 16/18 infection correlates well with vaccine efficacy against cervical HPV 16/18 infection (89.9%).^34^ A randomized clinical trial demonstrated that oral HPV prevalence 4 years after vaccination with bivalent vaccine was much lower among HPV vaccinated women.^35^ However, more population‐based evidence for the potential benefit of existing prophylactic https://www.sciencedirect.com/topics/medicine-and-dentistry/vaccine are needed. Lack of screening programs make the demand for preventive vaccine even more imperative for HPV‐attributable noncervical cancers. Cost‐effectiveness of HPV vaccination should be considered for both cervical cancer and other HPV‐attributable noncervical cancers. The largest noncervical cancer burden, both in terms of incidence and absolute numbers, was after 60 years of age both for males and females. Exploring accessible screening technologies for HPV‐attributable noncervical cancers should also be developed. Protection of vulnerable population such as HIV‐positive individuals and men who have sex with men would require effective implementation of gender‐neutral HPV vaccines and screenings.^36^, ^37^

Chinese males and females contributed 17.6% new cancer cases attributable to HPV infection in the world,^2^ which was constituted a high proportion of global HPV‐attributable cancers. Successful control of cancers attributable to HPV infection will help to fulfill the government‐approved "Healthy China 2030" goals to reduce premature mortality of major noncommunicable disease. Moreover, effective control of HPV attributable cancers is a key towards global cancer prevention, and our estimation will be a nice illustration to the low‐ and middle‐income countries.

Our study analyzed sex‐, cancer site‐, age‐, and geographical area‐ specific cancer burden attributable to HPV in China. Using the latest and most representative data of cancer incidence and mortality based on the published Chinese cancer registry annual report,^16^ we assessed HPV‐attributable cancer cases and deaths in all 31 provinces in mainland China, the registry data cover approximately 21% of the Chinese population. It is currently the first accurate and comprehensive estimation of HPV‐attributable cancer burden in China. Our study provides a national profile of current cancer burden attributable to HPV infection at a population level, supplies fundamental data and scientific evidences for policy‐making on HPV‐attributable cancer control and prevention. However, there are some limitations in the study. First, our results were estimated by using population attributable fractions from the international literature. The international PAFs may not be exactly the same with Chinese PAFs, which may cause an over‐ or under‐estimation of the actual cancer cases and deaths attributable to HPV. Secondly, our study evaluated all anal cancers regardless of pathological type, because the pathological specific rates are not available in the report of cancer registry. As such, we may overestimate the burden of anal cancer attributable to HPV infection because anal adenocarcinoma is not considered to be associated with HPV infection.

In summary, the cancer burden attributable to HPV in China was substantial: overall 110 894 new cancer cases and 35 683 cancer deaths per year were estimated and cervical cancer contributed the most new cases and deaths. HPV vaccination and cervical screening should be prioritized in China. Further estimation on the temporal trend, especially for oropharyngeal cancer and anal cancer are required in order to provide more precise estimation of HPV‐attributable cancer burden and to formulate recommendation on related cancer control.

## Data Availability

All datasets generated for this study are included in the manuscript or supplementary material.
