# Supplementary material for: Cancer burden attributable to human papillomavirus infection by sex, cancer site, age, and geographical area in China
Source: Cancer Med. 2019 Nov 12;9(1):374–84. doi: 10.1002/cam4.2697 (PMC6943148; doi:10.1002/cam4.2697)
Supplement: Supplementary file 1 [file CAM4-9-374-s001.docx]

**Supplementary appendix**

**Title: Cancer burden attributable to human papillomavirus infection by sex, cancer site, age and geographical area in China**

**Authors:** Rufei Duan, Youlin Qiao, Gary Clifford, Fanghui Zhao

**Content**

eTable 1. Cancer cases and incidence (per 100,000 persons) in HPV-associated cancer sites in China, 2014

eTable 2. Cancer cases and incidence (per 100,000 persons) attributable to HPV infection in China, 2014

eTable 3. Cancer deaths and mortality (per 100,000 persons) in HPV-associated cancer sites in China, 2014

eTable 4. Cancer deaths and mortality (per 100,000 persons) attributable to HPV infection in China, 2014

eFigure 1. Age-specific cancer cases in HPV-associated cancer sites in China, male, 2014

eFigure 2. Age-specific (A) all cancer cases and (B) non-cervical cancer cases in HPV-associated cancer sites in China, female, 2014

eFigure 3. Age-specific cancer deaths in HPV-associated cancer sites in China, male, 2014

eFigure 4. Age-specific (A) all cancer deaths and (B) non-cervical cancer deaths in HPV-associated cancer sites in China, female, 2014

eFigure 5. Age-specific cancer incidence in HPV-associated cancer sites in China, male, 2014

eFigure 6. Age-specific (A) all cancer incidence and (B) non-cervical cancer incidence in HPV-associated cancer sites in China, female, 2014

eFigure 7. Age-specific cancer mortality in HPV-associated cancer sites in China, male, 2014

eFigure 8. Age-specific (A) all cancer mortality (B) non-cervical cancer mortality in HPV-associated cancer sites in China, female, 2014

**eTable 1.** Cancer cases and incidence (per 100,000 persons) in HPV-associated cancer sites in China, 2014

| Cancer site (ICD-10) | **Total** | | |  | **Male** | | |  | **Female** | | |
| --- | --- | --- | --- | --- | --- | --- | --- | --- | --- | --- | --- |
|  | **Cases** | **CR** | **ASIR** |  | **Cases** | **CR** | **ASIR** |  | **Cases** | **CR** | **ASIR** |
| All areas |  |  |  |  |  |  |  |  |  |  |  |
| Cervix uteri (C53) | 102074 | 7.46 | 5.26 |  | .. | .. | .. |  | 102074 | 15.3 | 10.61 |
| Anus (C21) | 4493 | 0.33 | 0.21 |  | 2575 | 0.37 | 0.25 |  | 1918 | 0.29 | 0.18 |
| Vulva (C51) | 2789 | 0.20 | 0.13 |  | .. | .. | .. |  | 2789 | 0.42 | 0.26 |
| Vagina (C52) | 1601 | 0.12 | 0.08 |  | .. | .. | .. |  | 1601 | 0.24 | 0.16 |
| Penis (C60) | 4700 | 0.34 | 0.23 |  | 4700 | 0.67 | 0.46 |  | .. | .. | .. |
| Oropharynx (C01, 09-10) | 6018 | 0.44 | 0.30 |  | 4516 | 0.64 | 0.46 |  | 1503 | 0.23 | 0.15 |
| Oral cavity (C02-06) | 20831 | 1.52 | 1.02 |  | 12954 | 1.85 | 1.29 |  | 7876 | 1.18 | 0.76 |
| Larynx (C32) | 23408 | 1.71 | 1.14 |  | 20786 | 2.97 | 2.05 |  | 2622 | 0.39 | 0.24 |
| Other pharynx (C12-14) | 9118 | 0.67 | 0.45 |  | 7953 | 1.13 | 0.79 |  | 1165 | 0.17 | 0.11 |
| Total | 175032 | 12.79 | 8.82 |  | 53484 | 7.63 | 5.30 |  | 121548 | 18.22 | 12.47 |
| Urban areas |  |  |  |  |  |  |  |  |  |  |  |
| Cervix uteri (C53) | 56446 | 7.53 | 5.1 |  | .. | .. | .. |  | 56446 | 15.27 | 10.21 |
| Anus (C21) | 2328 | 0.31 | 0.19 |  | 1317 | 0.35 | 0.22 |  | 1011 | 0.27 | 0.16 |
| Vulva (C51) | 1793 | 0.24 | 0.14 |  | .. | .. | .. |  | 1793 | 0.49 | 0.28 |
| Vagina (C52) | 956 | 0.13 | 0.08 |  | .. | .. | .. |  | 956 | 0.26 | 0.17 |
| Penis (C60) | 2458 | 0.33 | 0.20 |  | 2458 | 0.65 | 0.41 |  | .. | .. | .. |
| Oropharynx (C01, 09-10) | 3770 | 0.50 | 0.33 |  | 2826 | 0.74 | 0.5 |  | 944 | 0.26 | 0.16 |
| Oral cavity (C02-06) | 13469 | 1.80 | 1.14 |  | 8362 | 2.20 | 1.44 |  | 5107 | 1.38 | 0.84 |
| Larynx (C32) | 14511 | 1.94 | 1.22 |  | 13175 | 3.47 | 2.26 |  | 1336 | 0.36 | 0.20 |
| Other pharynx (C12-14) | 5556 | 0.74 | 0.47 |  | 4941 | 1.30 | 0.85 |  | 615 | 0.17 | 0.10 |
| Total | 101287 | 13.52 | 8.87 |  | 33079 | 8.71 | 5.68 |  | 68208 | 18.46 | 12.12 |
| Rural areas |  |  |  |  |  |  |  |  |  |  |  |
| Cervix uteri (C53) | 45628 | 7.38 | 5.47 |  | .. | .. | .. |  | 45628 | 15.34 | 11.16 |
| Anus (C21) | 2165 | 0.35 | 0.25 |  | 1258 | 0.39 | 0.29 |  | 907 | 0.31 | 0.21 |
| Vulva (C51) | 996 | 0.16 | 0.12 |  | .. | .. | .. |  | 996 | 0.33 | 0.23 |
| Vagina (C52) | 645 | 0.10 | 0.08 |  | .. | .. | .. |  | 645 | 0.22 | 0.15 |
| Penis (C60) | 2242 | 0.36 | 0.26 |  | 2242 | 0.70 | 0.52 |  | .. | .. | .. |
| Oropharynx (C01, 09-10) | 2248 | 0.36 | 0.27 |  | 1690 | 0.53 | 0.41 |  | 558 | 0.19 | 0.13 |
| Oral cavity (C02-06) | 7362 | 1.19 | 0.87 |  | 4592 | 1.43 | 1.08 |  | 2770 | 0.93 | 0.65 |
| Larynx (C32) | 8897 | 1.44 | 1.03 |  | 7611 | 2.37 | 1.77 |  | 1286 | 0.43 | 0.29 |
| Other pharynx (C12-14) | 3562 | 0.58 | 0.42 |  | 3012 | 0.94 | 0.7 |  | 550 | 0.18 | 0.13 |
| Total | 73745 | 11.92 | 8.77 |  | 20405 | 6.36 | 4.77 |  | 53340 | 17.93 | 12.95 |

Abbreviations: ICD-10, International Classification of Diseases 10th revision; CR, crude rate; ASIR, age-standardized incidence rate.

**eTable 2.** Cancer cases and incidence (per 100,000 persons) attributable to HPV infection in China, 2014

| Cancer site (ICD-10) | **Total** | | | | **Male** | | | **Female** | | |
| --- | --- | --- | --- | --- | --- | --- | --- | --- | --- | --- |
|  | **Cases** | **PAF**  **(%)** | **Cases attributable to HPV (95% CI)** | **ASIR** | **Cases** | **Cases**  **attributable**  **to HPV (95% CI)** | **ASIR** | **Cases** | **Cases**  **attributable**  **to HPV (95% CI)** | **ASIR** |
| All areas |  |  |  |  |  |  |  |  |  |  |
| Cervix uteri (C53) | 102074 | 97.4 | 99253 (99214–99293) | 5.15 | .. | .. | .. | 102074 | 99253 (99214–99293) | 10.42 |
| Anus (C21) | 4493 | 88.0 | 3936 (3802–4070) | 0.17 | 2575 | 2258 (2181–2335) | 0.20 | 1918 | 1678 (1621–1735) | 0.15 |
| Vulva (C51) | 2789 | 24.1 | 736 (604–857) | 0.04 | .. | .. | .. | 2789 | 736 (604–857) | 0.07 |
| Vagina (C52) | 1601 | 78.0 | 1237 (1079–1364) | 0.06 | .. | .. | .. | 1601 | 1237 (1079–1364) | 0.12 |
| Penis (C60) | 4700 | 51.0 | 2392 (2204–2579) | 0.11 | 4700 | 2392 (2204–2579) | 0.22 | .. | .. | .. |
| Oropharynx (C01, 09-10) | 6018 | 23.0 | 1374 (1016–1613) | 0.07 | 4516 | 1032 (763–1212) | 0.10 | 1503 | 342 (253–401) | 0.03 |
| Oral cavity (C02-06) | 20831 | 4.3 | 891 (663–1181) | 0.04 | 12954 | 555 (413–736) | 0.05 | 7876 | 336 (250–445) | 0.03 |
| Larynx (C32) | 23408 | 4.6 | 1075 (771–1426) | 0.05 | 20786 | 955 (685–1266) | 0.09 | 2622 | 120 (86–160) | 0.01 |
| Total | **165914** | **..** | **110894** | **5.69** | **45531** | **7192** | **0.66** | **120383** | **103702** | **10.83** |
| Urban areas |  |  |  |  |  |  |  |  |  |  |
| Cervix uteri (C53) | 56446 | 97.4 | 54875 (54854–54898) | 4.99 | .. | .. | .. | 56446 | 54875 (54854–54898) | 10.01 |
| Anus (C21) | 2328 | 88.0 | 2043 (1973–2113) | 0.15 | 1317 | 1159 (1119–1198) | 0.18 | 1011 | 884 (854–914) | 0.13 |
| Vulva (C51) | 1793 | 24.1 | 450 (367–525) | 0.04 | .. | .. | .. | 1793 | 450 (367–525) | 0.07 |
| Vagina (C52) | 956 | 78.0 | 737 (643–813) | 0.06 | .. | .. | .. | 956 | 737 (643–813) | 0.13 |
| Penis (C60) | 2458 | 51.0 | 1251 (1152–1349) | 0.10 | 2458 | 1251 (1152–1349) | 0.19 | .. | .. | .. |
| Oropharynx (C01, 09-10) | 3770 | 23.0 | 863 (638–1013) | 0.07 | 2826 | 649 (479–761) | 0.11 | 944 | 214 (158–252) | 0.03 |
| Oral cavity (C02-06) | 13469 | 4.3 | 577 (429–764) | 0.05 | 8362 | 359 (267–476) | 0.06 | 5107 | 218 (162–289) | 0.03 |
| Larynx (C32) | 14511 | 4.6 | 667 (478–884) | 0.06 | 13175 | 605 (434–803) | 0.10 | 1336 | 61 (44–81) | 0.01 |
| Total | **95731** | **..** | **61463** | **5.52** | **28138** | **4023** | **0.64** | **67593** | **57439** | **10.41** |
| Rural areas |  |  |  |  |  |  |  |  |  |  |
| Cervix uteri (C53) | 45628 | 97.4 | 44377 (44360–44395) | 5.37 | .. | .. | .. | 45628 | 44377 (44360–44395) | 10.99 |
| Anus (C21) | 2165 | 88.0 | 1893 (1829–1958) | 0.20 | 1258 | 1099 (1062–1137) | 0.24 | 907 | 794 (767–821) | 0.17 |
| Vulva (C51) | 996 | 24.1 | 286 (238–332) | 0.03 | .. | .. | .. | 996 | 286 (238–332) | 0.07 |
| Vagina (C52) | 645 | 78.0 | 500 (436–551) | 0.06 | .. | .. | .. | 645 | 500 (436–551) | 0.12 |
| Penis (C60) | 2242 | 51.0 | 1141 (1052–1230) | 0.13 | 2242 | 1141 (1052–1230) | 0.25 | .. | .. | .. |
| Oropharynx (C01, 09-10) | 2248 | 23.0 | 511 (378–600) | 0.06 | 1690 | 384 (284–451) | 0.09 | 558 | 128 (94–150) | 0.03 |
| Oral cavity (C02-06) | 7362 | 4.30 | 314 (234–416) | 0.04 | 4592 | 196 (146–260) | 0.05 | 2770 | 118 (88–156) | 0.03 |
| Larynx (C32) | 8897 | 4.60 | 409 (293–542) | 0.05 | 7611 | 350 (251–464) | 0.08 | 1286 | 59 (42–78) | 0.01 |
| Total | **70183** | **..** | **49431** | **5.94** | **17393** | **3170** | **0.71** | **52790** | **46262** | **11.42** |

Abbreviations: ICD-10, International Classification of Diseases 10th revision; PAF, population attributable fraction; CI, Confidence interval; ASIR, age-standardized incidence rate.

**eTable 3.** Cancer deaths and mortality (per 100,000 persons) HPV-associated cancer sites in China, 2014

| Cancer site (ICD-10) | **Total** | | |  | **Male** | | |  | **Female** | | |
| --- | --- | --- | --- | --- | --- | --- | --- | --- | --- | --- | --- |
|  | **Cases** | **CR** | **ASMR** |  | **Cases** | **CR** | **ASMR** |  | **Cases** | **CR** | **ASMR** |
| All areas |  |  |  |  |  |  |  |  |  |  |  |
| Cervix uteri (C53) | 30464 | 2.23 | 1.49 |  | .. | .. | .. |  | 30464 | 4.57 | 2.98 |
| Anus (C21) | 3157 | 0.23 | 0.14 |  | 1849 | 0.26 | 0.17 |  | 1308 | 0.20 | 0.11 |
| Vulva (C51) | 1043 | 0.08 | 0.05 |  | .. | .. | .. |  | 1043 | 0.16 | 0.09 |
| Vagina (C52) | 693 | 0.05 | 0.03 |  | .. | .. | .. |  | 693 | 0.1 | 0.07 |
| Penis (C60) | 1460 | 0.11 | 0.07 |  | 1460 | 0.21 | 0.14 |  | .. | .. | .. |
| Oropharynx (C01, 09-10) | 2825 | 0.21 | 0.13 |  | 2222 | 0.32 | 0.22 |  | 603 | 0.09 | 0.05 |
| Oral cavity (C02-06) | 10340 | 0.76 | 0.48 |  | 6627 | 0.95 | 0.64 |  | 3713 | 0.56 | 0.32 |
| Larynx (C32) | 13201 | 0.97 | 0.60 |  | 11509 | 1.64 | 1.08 |  | 1692 | 0.25 | 0.14 |
| Other pharynx (C12-14) | 5232 | 0.38 | 0.25 |  | 4493 | 0.64 | 0.44 |  | 739 | 0.11 | 0.06 |
| Total | 68415 | 5.02 | 3.24 |  | 28160 | 4.02 | 2.69 |  | 40255 | 6.04 | 3.82 |
| Urban areas |  |  |  |  |  |  |  |  |  |  |  |
| Cervix uteri (C53) | 16429 | 2.19 | 1.40 |  | .. | .. | .. |  | 16429 | 4.44 | 2.78 |
| Anus (C21) | 1764 | 0.24 | 0.14 |  | 1044 | 0.28 | 0.17 |  | 720 | 0.19 | 0.10 |
| Vulva (C51) | 685 | 0.09 | 0.05 |  | .. | .. | .. |  | 685 | 0.19 | 0.10 |
| Vagina (C52) | 441 | 0.06 | 0.03 |  | .. | .. | .. |  | 441 | 0.12 | 0.07 |
| Penis (C60) | 770 | 0.10 | 0.06 |  | 770 | 0.20 | 0.12 |  | .. | .. | .. |
| Oropharynx (C01, 09-10) | 1752 | 0.23 | 0.14 |  | 1409 | 0.37 | 0.24 |  | 343 | 0.09 | 0.05 |
| Oral cavity (C02-06) | 6655 | 0.89 | 0.52 |  | 4302 | 1.13 | 0.72 |  | 2353 | 0.64 | 0.34 |
| Larynx (C32) | 7827 | 1.04 | 0.60 |  | 7009 | 1.85 | 1.13 |  | 818 | 0.22 | 0.11 |
| Other pharynx (C12-14) | 3277 | 0.44 | 0.27 |  | 2852 | 0.75 | 0.48 |  | 425 | 0.11 | 0.06 |
| Total | 39600 | 5.28 | 3.21 |  | 17386 | 4.58 | 2.86 |  | 22214 | 6.00 | 3.61 |
| Rural areas |  |  |  |  |  |  |  |  |  |  |  |
| Cervix uteri (C53) | 14035 | 2.27 | 1.63 |  | .. | .. | .. |  | 14035 | 4.72 | 3.27 |
| Anus (C21) | 1393 | 0.23 | 0.15 |  | 805 | 0.25 | 0.18 |  | 588 | 0.20 | 0.12 |
| Vulva (C51) | 358 | 0.06 | 0.04 |  | .. | .. | .. |  | 358 | 0.12 | 0.08 |
| Vagina (C52) | 252 | 0.04 | 0.03 |  | .. | .. | .. |  | 252 | 0.08 | 0.06 |
| Penis (C60) | 690 | 0.11 | 0.08 |  | 690 | 0.21 | 0.15 |  | .. | .. | .. |
| Oropharynx (C01, 09-10) | 1073 | 0.17 | 0.12 |  | 813 | 0.25 | 0.19 |  | 260 | 0.09 | 0.06 |
| Oral cavity (C02-06) | 3685 | 0.60 | 0.41 |  | 2325 | 0.72 | 0.53 |  | 1360 | 0.46 | 0.29 |
| Larynx (C32) | 5374 | 0.87 | 0.59 |  | 4500 | 1.40 | 1.01 |  | 874 | 0.29 | 0.18 |
| Other pharynx (C12-14) | 1955 | 0.32 | 0.22 |  | 1641 | 0.51 | 0.38 |  | 314 | 0.11 | 0.06 |
| Total | 28815 | 4.67 | 3.27 |  | 10774 | 3.34 | 2.44 |  | 18041 | 6.07 | 4.12 |

Abbreviations: ICD-10, International Classification of Diseases 10th revision; CR, crude rate; ASMR, age-standardized mortality rate.

**eTable 4.** Cancer deaths and mortality (per 100,000 persons) attributable to HPV infection in China, 2014

| Cancer site (ICD-10) | **Total** | | | | **Male** | | | **Female** | | |
| --- | --- | --- | --- | --- | --- | --- | --- | --- | --- | --- |
|  | **Deaths** | **PAF (%)** | **Deaths attributable to HPV (95% CI)** | **ASMR** | **Deaths** | **Deaths attributable to HPV (95% CI)** | **ASMR** | **Deaths** | **Deaths attributable to HPV (95% CI)** | **ASMR** |
| All areas |  |  |  |  |  |  |  |  |  |  |
| Cervix uteri (C53) | 30464 | 97.4 | 29683 (29673–29694) | 1.41 | .. | .. | .. | 30464 | 29683 (29673–29694) | 2.84 |
| Anus (C21) | 3157 | 88.0 | 2773 (2679–2868) | 0.11 | 1849 | 1623 (1568–1678) | 0.13 | 1308 | 1150 (1111–1190) | 0.08 |
| Vulva (C51) | 1043 | 24.1 | 248 (201–289) | 0.01 | .. | .. | .. | 1043 | 248 (201–289) | 0.02 |
| Vagina (C52) | 693 | 78.0 | 533 (465–588) | 0.02 | .. | .. | .. | 693 | 533 (465–588) | 0.04 |
| Penis (C60) | 1460 | 51.0 | 745 (686–803) | 0.03 | 1460 | 745 (686–803) | 0.06 | .. | .. | .. |
| Oropharynx (C01, 09-10) | 2825 | 23.0 | 650 (480–762) | 0.03 | 2222 | 511 (378–600) | 0.05 | 603 | 139 (102–163) | 0.01 |
| Oral cavity (C02-06) | 10340 | 4.3 | 444 (330–589) | 0.02 | 6627 | 285 (212–378) | 0.03 | 3713 | 159 (118–211) | 0.01 |
| Larynx (C32) | 13201 | 4.6 | 607 (436–805) | 0.03 | 11509 | 529 (380–702) | 0.05 | 1692 | 78 (56–103) | 0.01 |
| Total | **63183** | **..** | **35683** | **1.66** | **23667** | **3693** | **0.32** | **39516** | **31990** | **3.01** |
| Urban areas |  |  |  |  |  |  |  |  |  |  |
| Cervix uteri (C53) | 16429 | 97.4 | 16003 (15998–16009) | 1.31 | .. | .. | .. | 16429 | 16003 (15998–16009) | 2.63 |
| Anus (C21) | 1764 | 88.0 | 1548 (1495–1600) | 0.10 | 1044 | 914 (883–945) | 0.12 | 720 | 633 (612–655) | 0.08 |
| Vulva (C51) | 685 | 24.1 | 155 (124–181) | 0.01 | .. | .. | .. | 685 | 155 (124–181) | 0.02 |
| Vagina (C52) | 441 | 78.0 | 344 (300–379) | 0.02 | .. | .. | .. | 441 | 344 (300–379) | 0.04 |
| Penis (C60) | 770 | 51.0 | 393 (362–424) | 0.03 | 770 | 393 (362–424) | 0.05 | .. | .. | .. |
| Oropharynx (C01, 09-10) | 1752 | 23.0 | 403 (298–473) | 0.03 | 1409 | 324 (240–381) | 0.05 | 343 | 79 (58–92) | 0.01 |
| Oral cavity (C02-06) | 6655 | 4.3 | 286 (213–379) | 0.02 | 4302 | 185 (138–245) | 0.03 | 2353 | 101 (75–134) | 0.01 |
| Larynx (C32) | 7827 | 4.6 | 360 (258–477) | 0.02 | 7009 | 322 (231–428) | 0.05 | 818 | 38 (27–50) | 0.00 |
| Total | **36323** | **..** | **19492** | **1.54** | **14534** | **2138** | **0.30** | **21789** | **17353** | **2.79** |
| Rural areas |  |  |  |  |  |  |  |  |  |  |
| Cervix uteri (C53) | 14035 | 97.4 | 13680 (13675–13685) | 1.54 | .. | .. | .. | 14035 | 13680 (13675–13685) | 3.14 |
| Anus (C21) | 1393 | 88.0 | 1226 (1184–1267) | 0.11 | 805 | 709 (684–733) | 0.13 | 588 | 517 (499–535) | 0.09 |
| Vulva (C51) | 358 | 24.1 | 93 (77–108) | 0.01 | .. | .. | .. | 358 | 93 (77–108) | 0.02 |
| Vagina (C52) | 252 | 78.0 | 189 (165–209) | 0.02 | .. | .. | .. | 252 | 189 (165–209) | 0.04 |
| Penis (C60) | 690 | 51.0 | 352 (324–380) | 0.03 | 690 | 352 (324–380) | 0.07 | .. | .. | .. |
| Oropharynx (C01, 09-10) | 1073 | 23.0 | 247 (182–290) | 0.03 | 813 | 187 (138–219) | 0.04 | 260 | 60 (44–70) | 0.01 |
| Oral cavity (C02-06) | 3685 | 4.3 | 158 (118–210) | 0.02 | 2325 | 100 (74–132) | 0.02 | 1360 | 58 (44–78) | 0.01 |
| Larynx (C32) | 5374 | 4.6 | 247 (177–328) | 0.03 | 4500 | 207 (148–274) | 0.04 | 874 | 40 (29–53) | 0.01 |
| Total | **26860** | **..** | **16192** | **1.79** | **9133** | **1555** | **0.30** | **17727** | **14637** | **3.32** |

Abbreviations: ICD-10, International Classification of Diseases 10th revision; PAF, population attributable fraction; CI, Confidence interval; ASMR, age-standardized mortality rate.


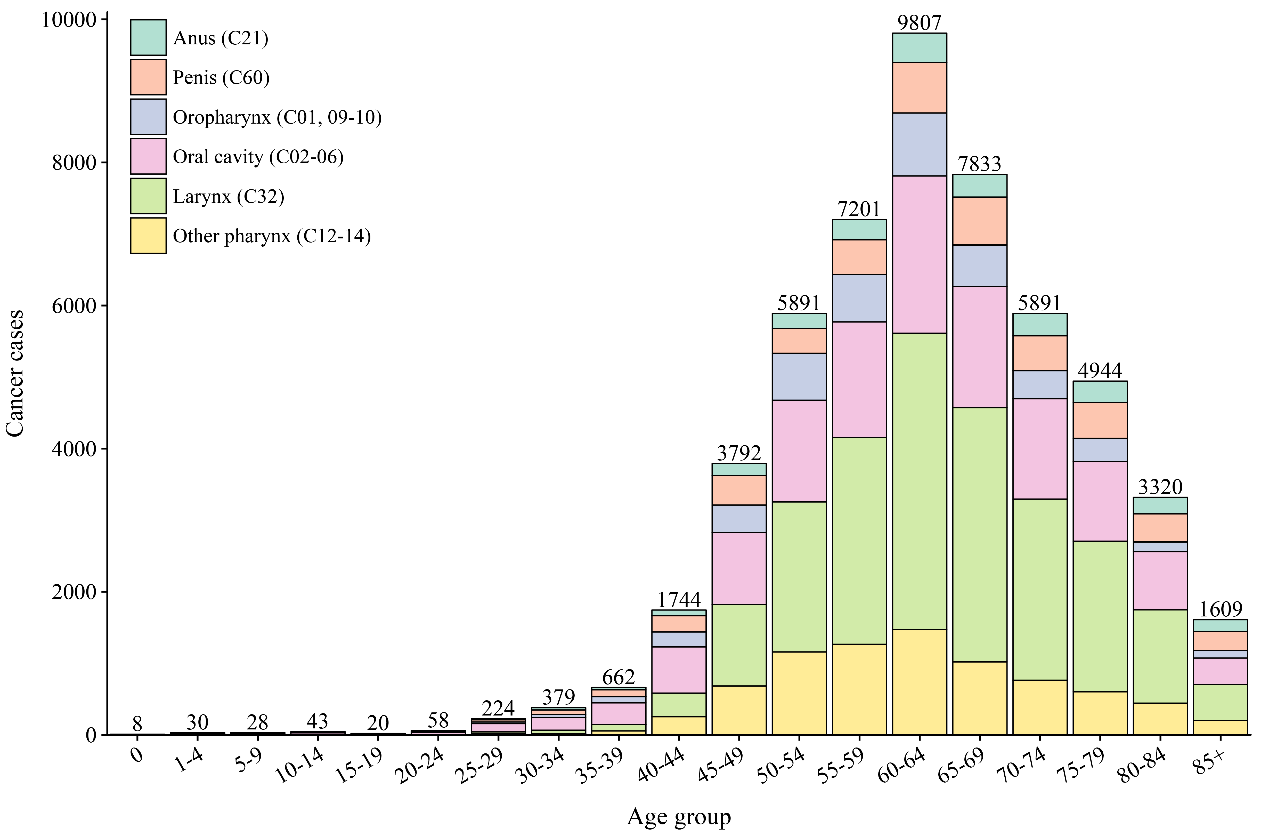


**eFigure 1.** Age-specific cancer cases in HPV-associated cancer sites in China, male, 2014


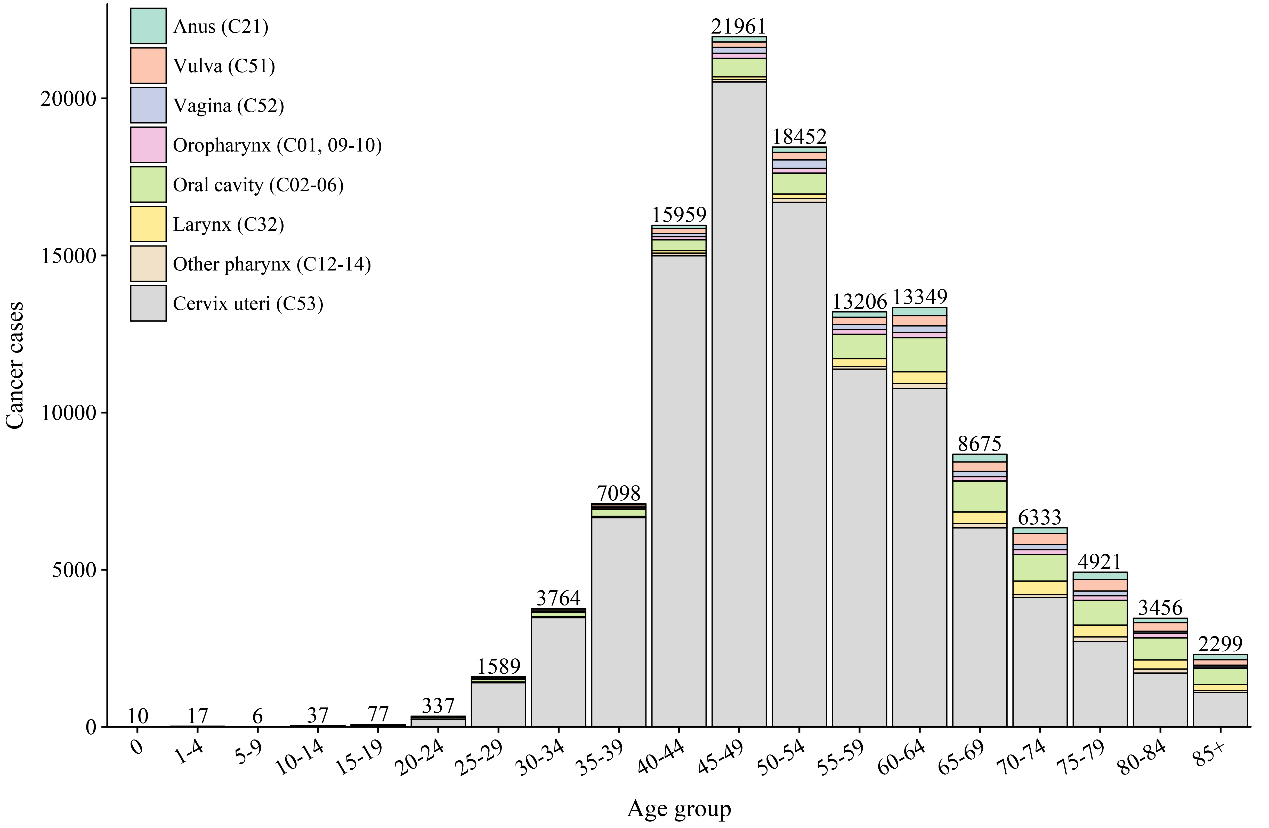


A: all cancer cases


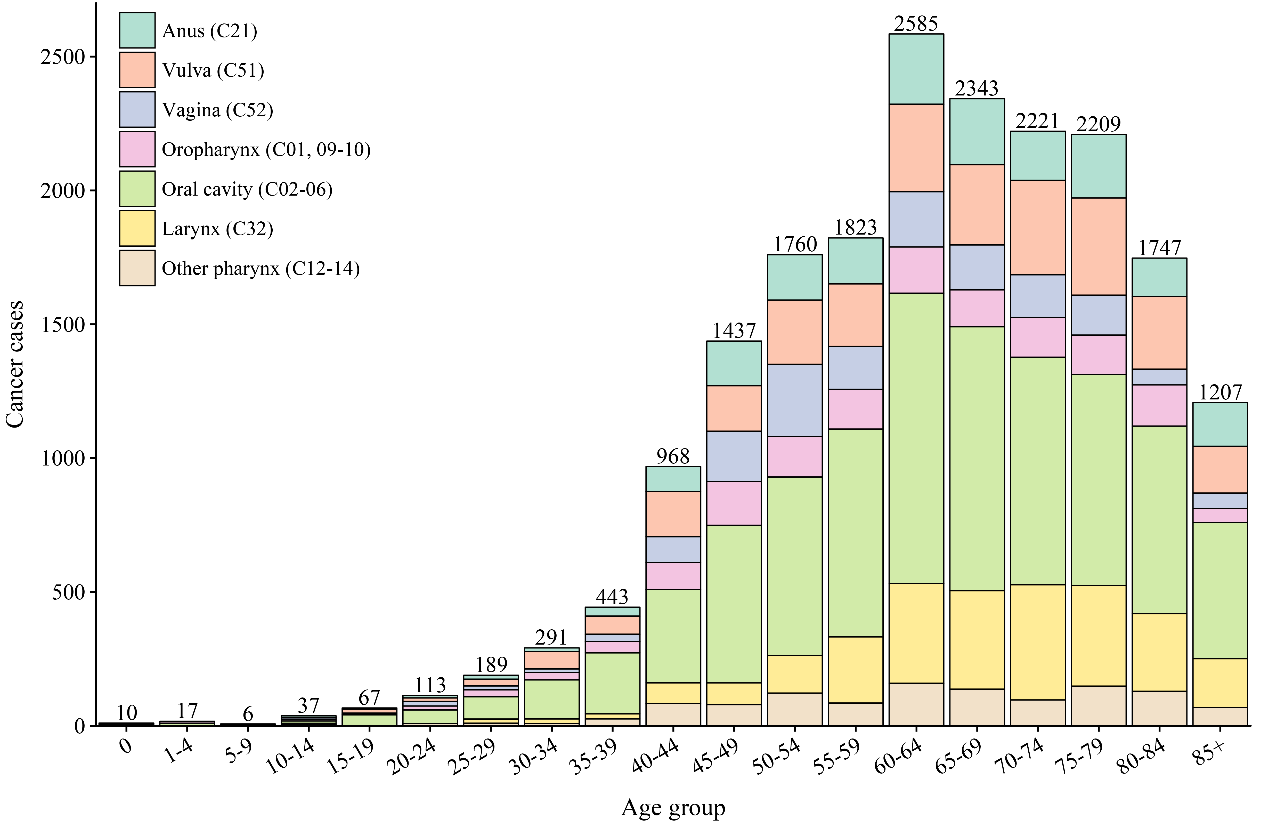


B: non-cervical cancer cases

**eFigure 2.** Age-specific (A) all cancer cases and (B) non-cervical cancer cases in HPV-associated cancer sites in China, female, 2014


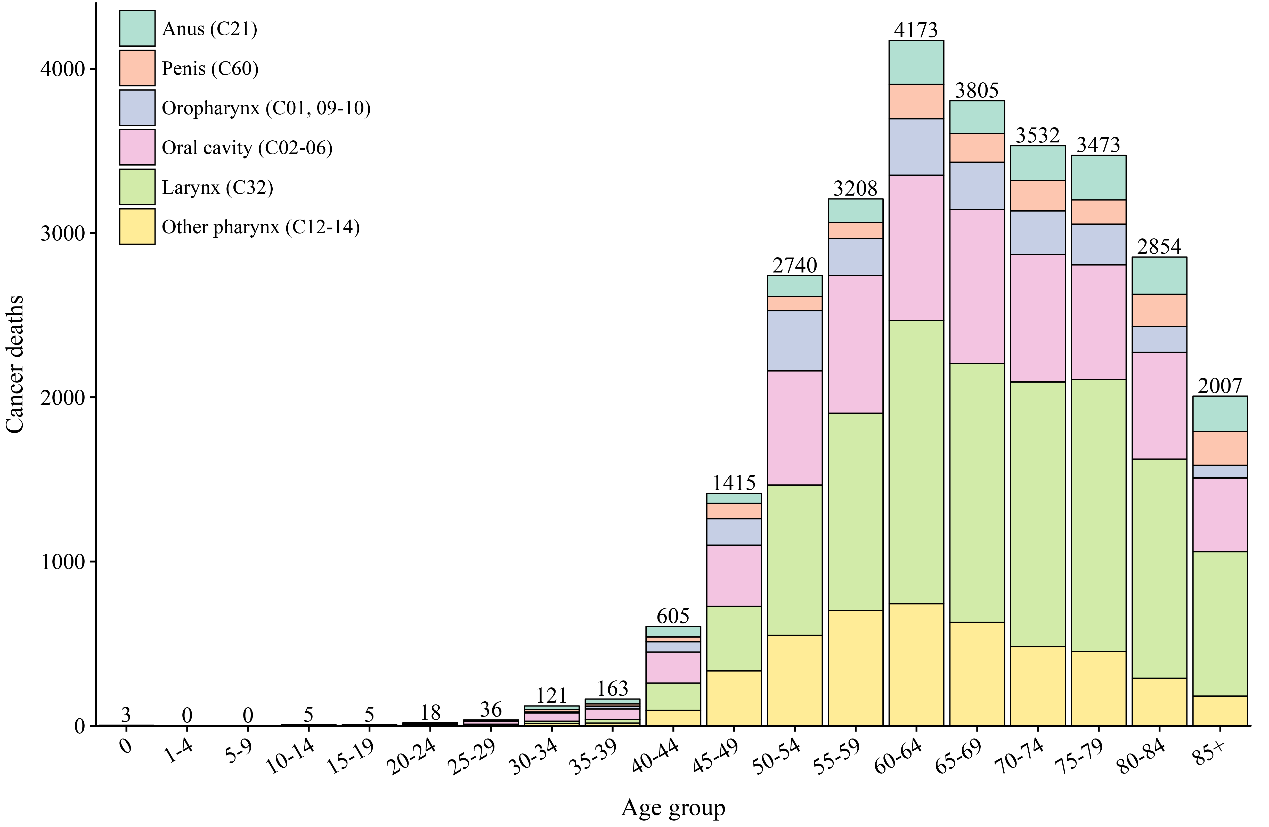


**eFigure 3.** Age-specific cancer deaths in HPV-associated cancer sites in China, male, 2014


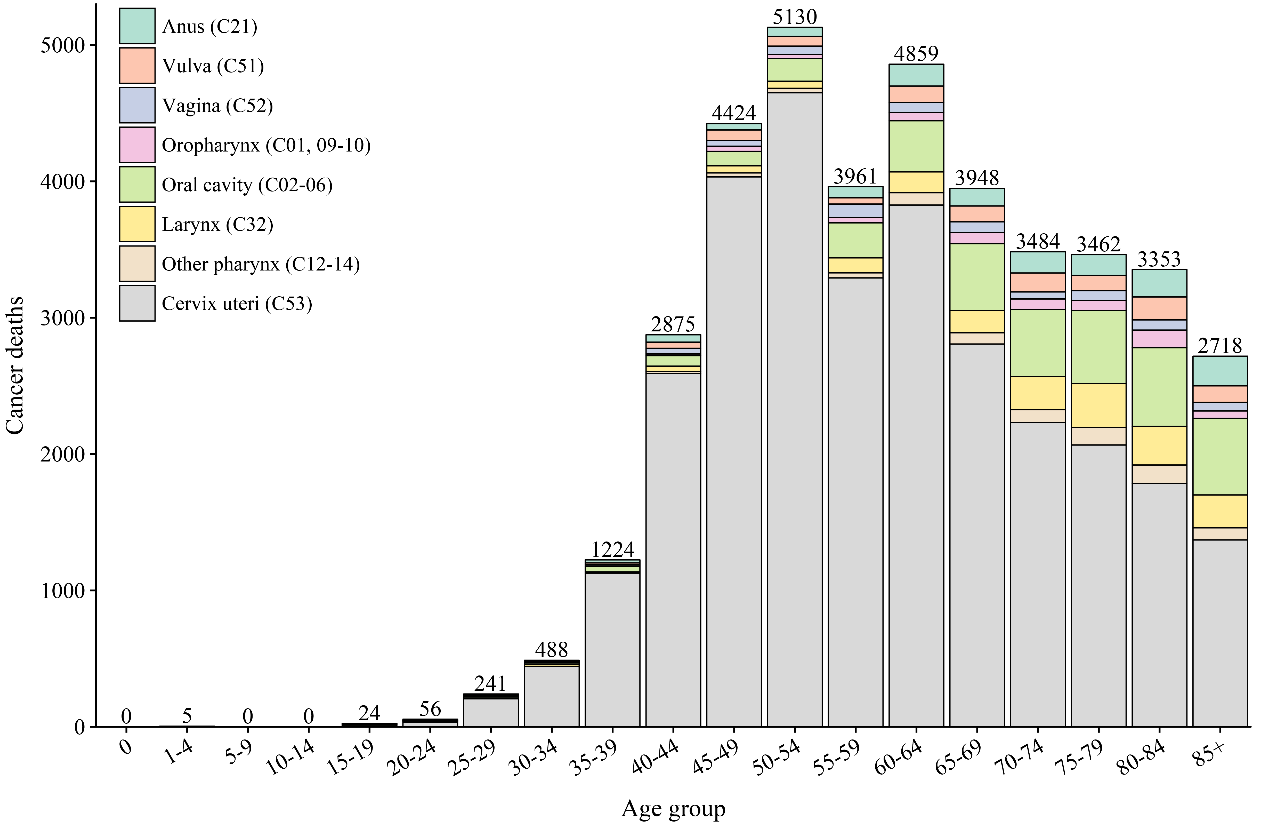


A: all cancer deaths


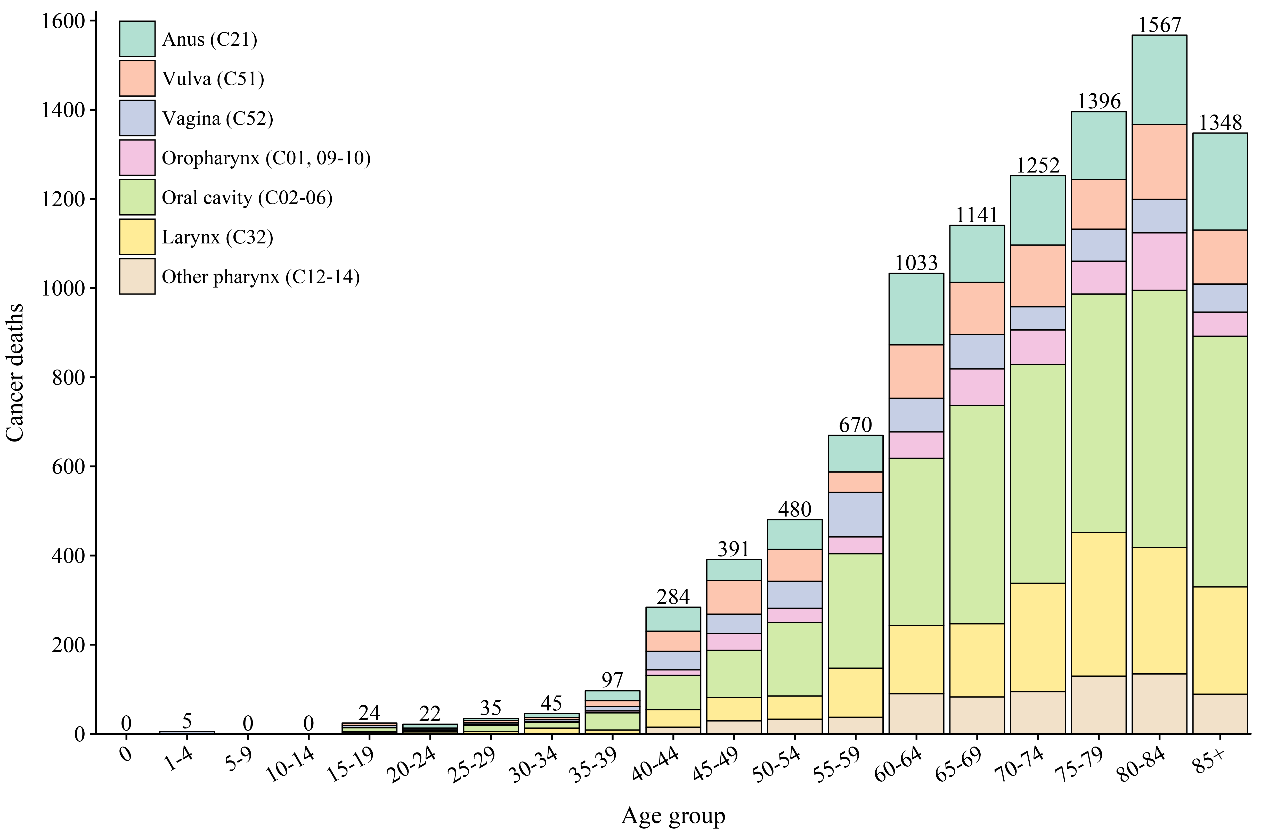


B: non-cervical cancer deaths

**eFigure 4.** Age-specific (A) all cancer deaths and (B) non-cervical cancer deaths in HPV-associated cancer sites in China, female, 2014


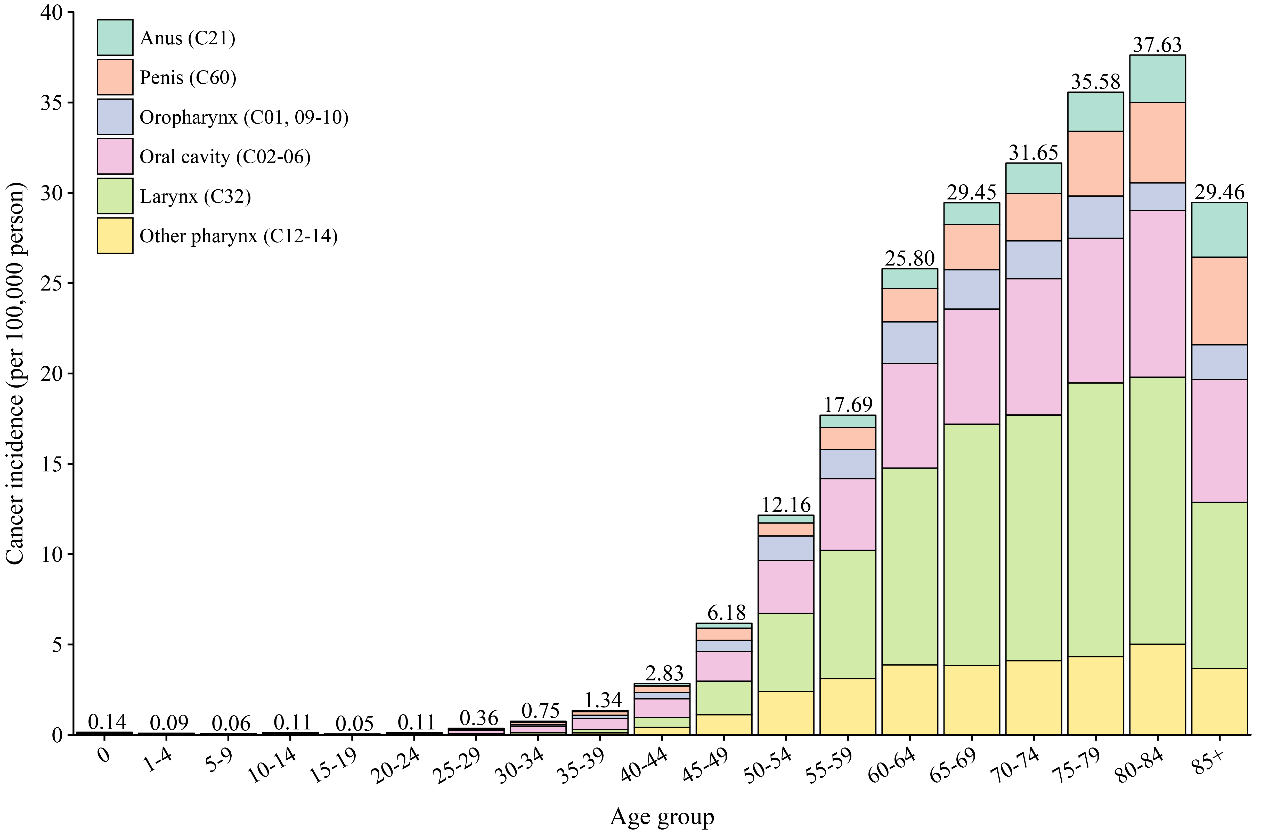


**eFigure 5.** Age-specific cancer incidence in HPV-associated cancer sites in China, male, 2014


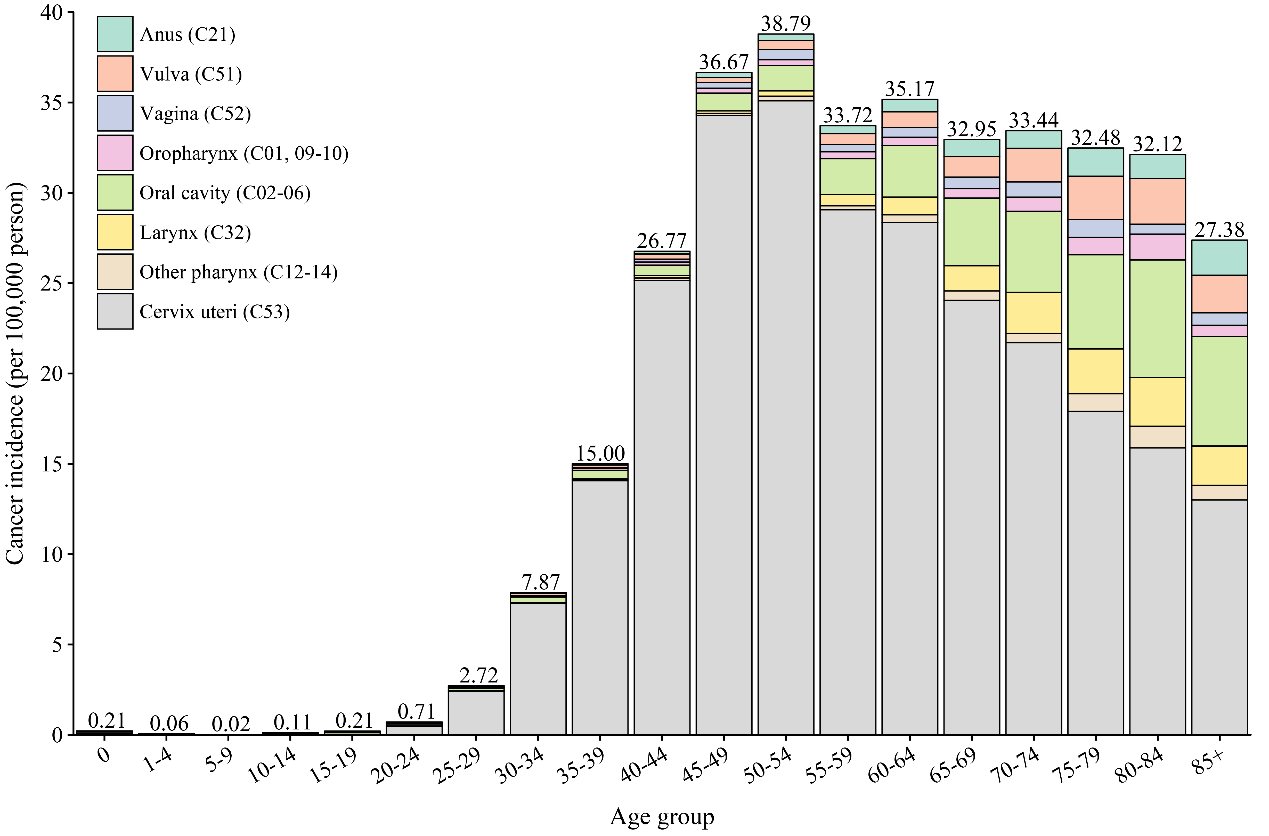


A: all cancer incidence


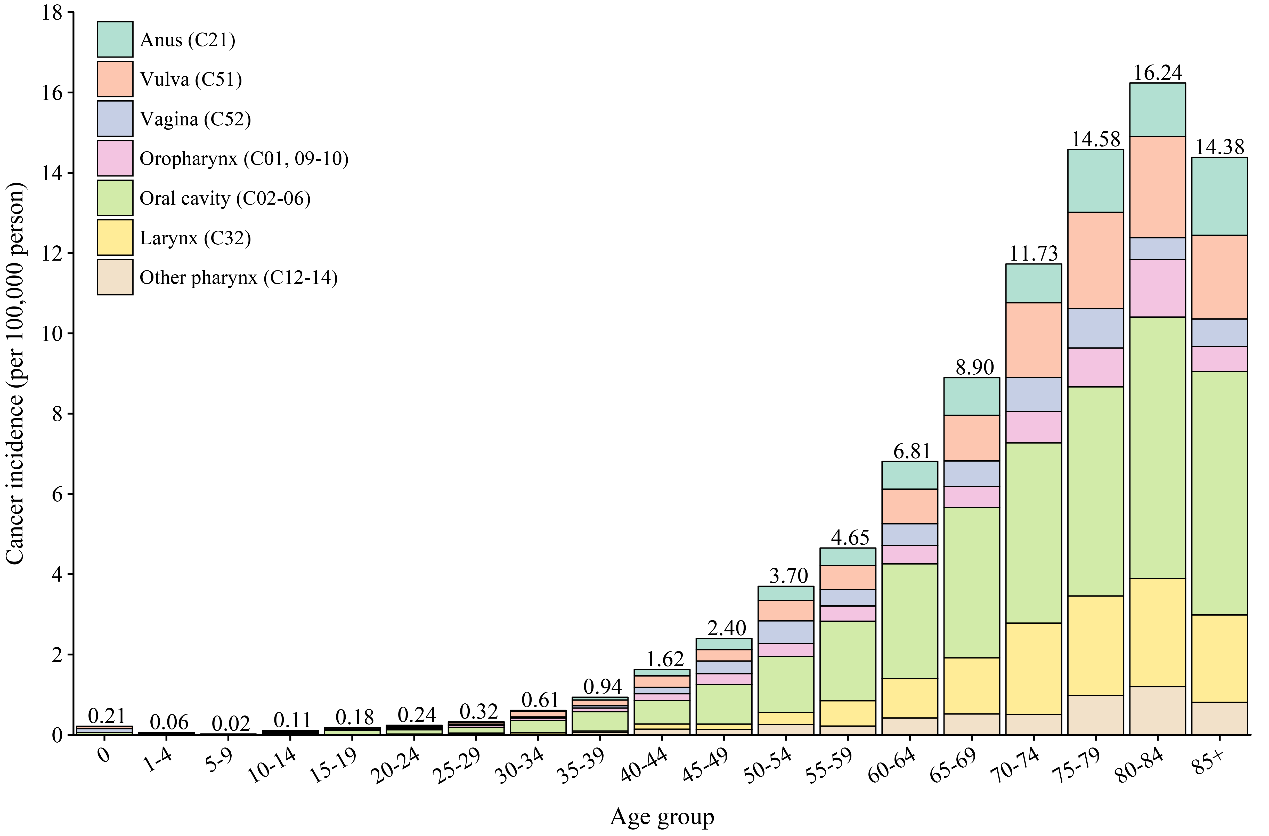


B: non-cervical cancer incidence

**eFigure 6.** Age-specific (A) all cancer incidence and (B) non-cervical cancer incidence in HPV-associated cancer sites in China, female, 2014


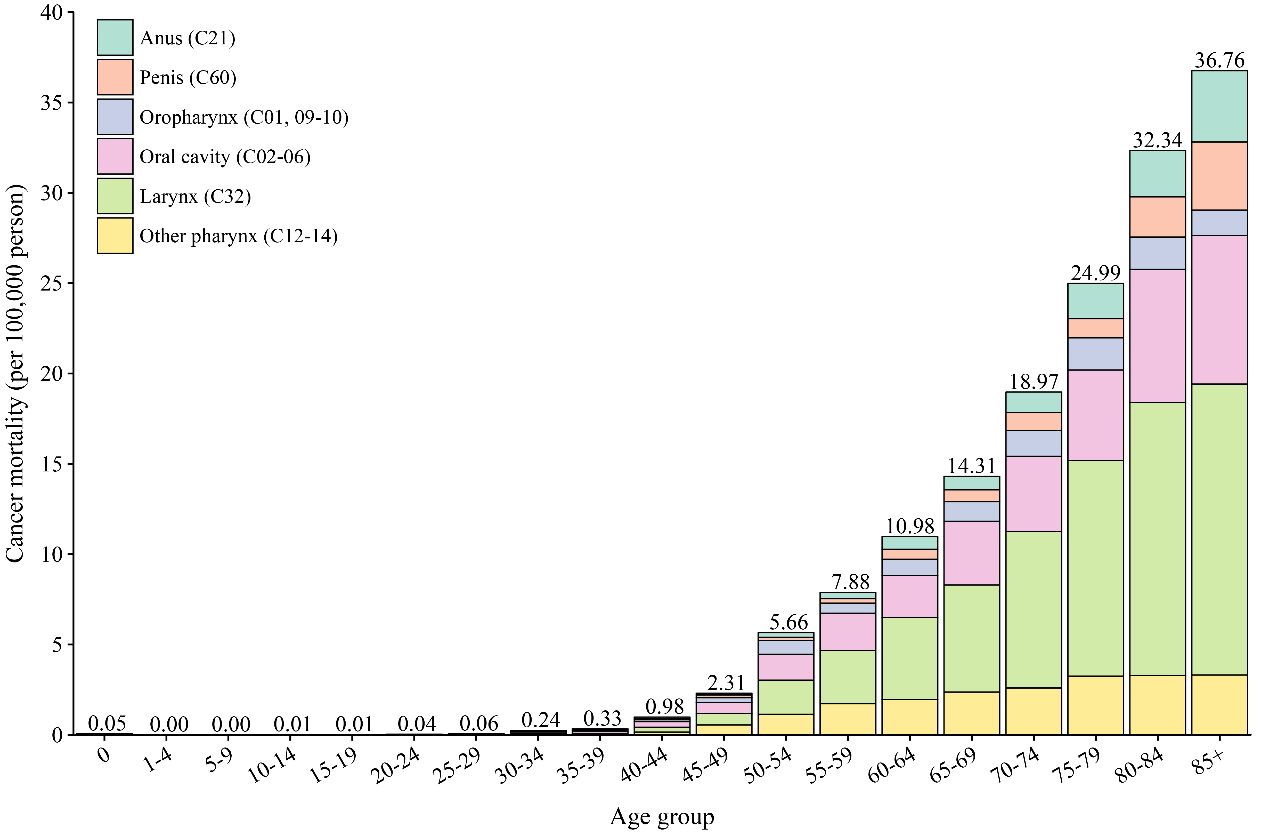


**eFigure 7.** Age-specific cancer mortality in HPV-associated cancer sites in China, male, 2014


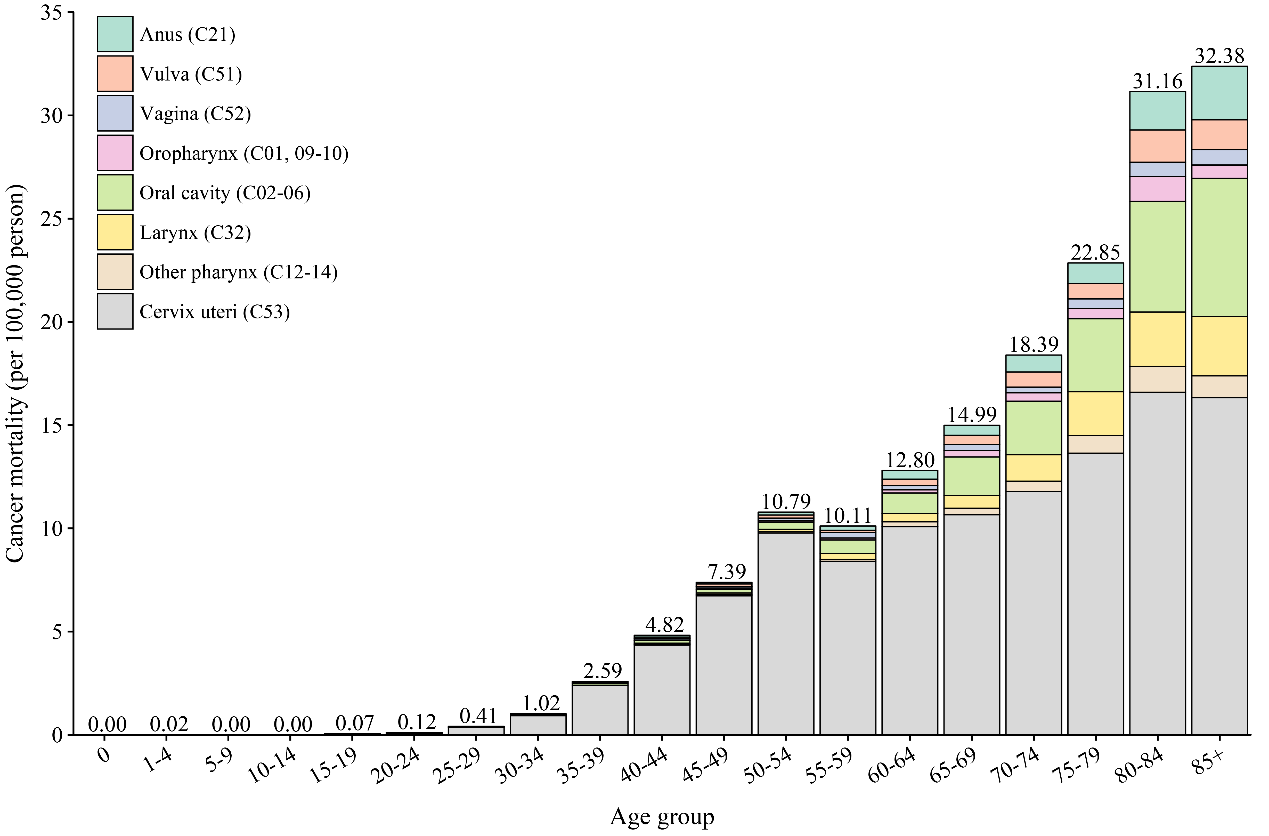


A: all cancer mortality


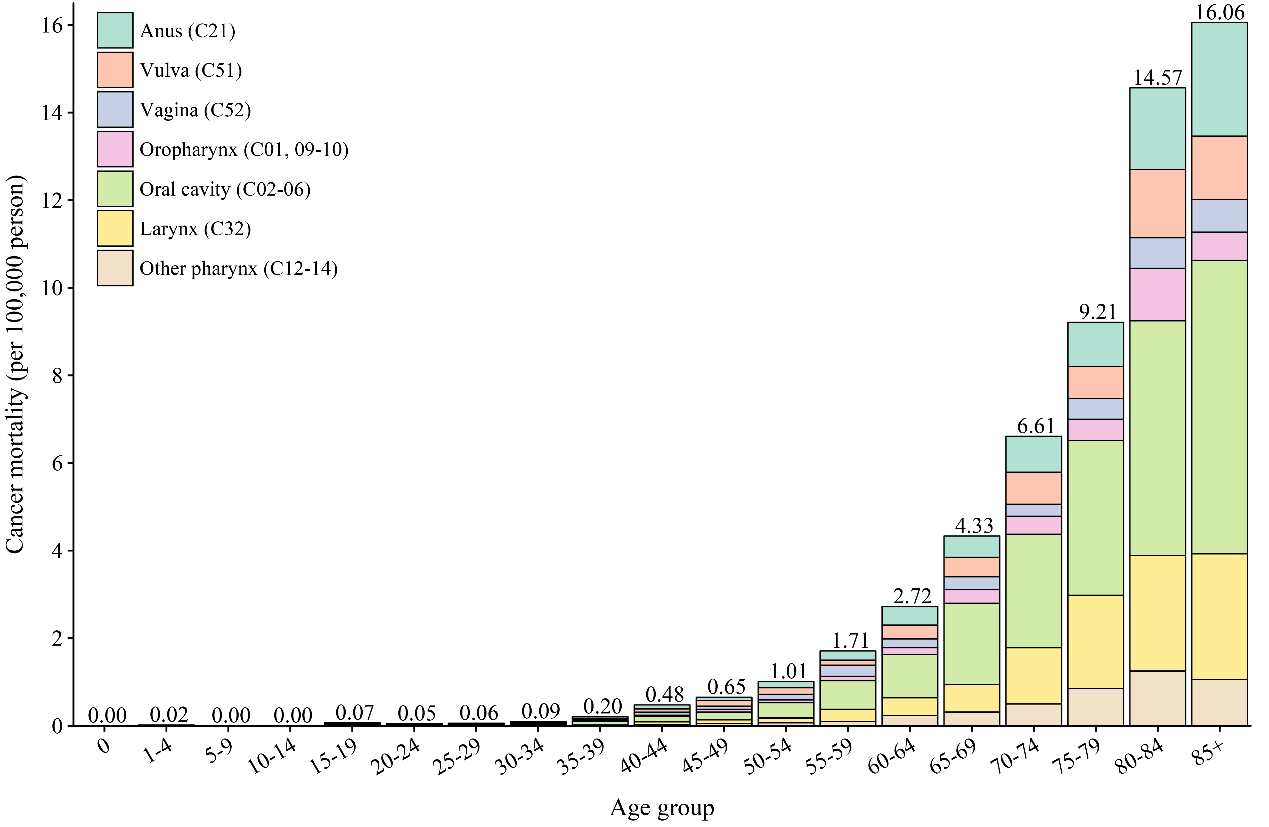


B: non-cervical cancer mortality

**eFigure 8.** Age-specific (A) all cancer mortality (B) non-cervical cancer mortality in HPV-associated cancer sites in China, female, 2014
